# Detection and significance of neuronal autoantibodies in patients with meningoencephalitis in Vientiane, Lao PDR

**DOI:** 10.1093/trstmh/trac023

**Published:** 2022-04-06

**Authors:** Christopher E Uy, Mayfong Mayxay, Ruby Harrison, Adam Al-Diwani, Leslie Jacobson, Sayaphet Rattanavong, Audrey Dubot-Pérès, Manivanh Vongsouvath, Viengmon Davong, Vilada Chansamouth, Koukeo Phommasone, Patrick Waters, Sarosh R Irani, Paul N Newton

**Affiliations:** Oxford Autoimmune Neurology Group, Nuffield Department of Clinical Neurosciences, Nuffield Department of Medicine, Oxford University, John Radcliffe Hospital, Oxford OX3 9DU, UK; Division of Neurology, Department of Medicine, University of British Columbia Hospital, Vancouver, British Columbia V6T 2B5, Canada; Department of Neurology, Oxford University Hospitals, John Radcliffe Hospital, Oxford OX3 9DU, UK; Lao-Oxford-Mahosot Hospital-Wellcome Research Unit OX3 7JX (LOMWRU), Microbiology Laboratory, Mahosot Hospital, Vientiane, Lao PDR; Center for Tropical Medicine and Global Health, Nuffield Department of Medicine, New Richards Building, Oxford University, Oxford OX3 7LG, UK; Institute of Research and Education Development (IRED), University of Health Sciences, Ministry of Health, Vientiane, Lao PDR; Oxford Autoimmune Neurology Group, Nuffield Department of Clinical Neurosciences, Nuffield Department of Medicine, Oxford University, John Radcliffe Hospital, Oxford OX3 9DU, UK; Oxford Autoimmune Neurology Group, Nuffield Department of Clinical Neurosciences, Nuffield Department of Medicine, Oxford University, John Radcliffe Hospital, Oxford OX3 9DU, UK; Department of Psychiatry, Warneford Hospital, University of Oxford, Oxford, UK; Oxford Autoimmune Neurology Group, Nuffield Department of Clinical Neurosciences, Nuffield Department of Medicine, Oxford University, John Radcliffe Hospital, Oxford OX3 9DU, UK; Lao-Oxford-Mahosot Hospital-Wellcome Research Unit OX3 7JX (LOMWRU), Microbiology Laboratory, Mahosot Hospital, Vientiane, Lao PDR; Lao-Oxford-Mahosot Hospital-Wellcome Research Unit OX3 7JX (LOMWRU), Microbiology Laboratory, Mahosot Hospital, Vientiane, Lao PDR; Center for Tropical Medicine and Global Health, Nuffield Department of Medicine, New Richards Building, Oxford University, Oxford OX3 7LG, UK; Unité des Virus Émergents (UVE: Aix-Marseille Univ-IRD 190-INSERM 1207), IHU Méditerranée Infection, 19-21, Bd Jean Moulin, Marseille 13005, France; Lao-Oxford-Mahosot Hospital-Wellcome Research Unit OX3 7JX (LOMWRU), Microbiology Laboratory, Mahosot Hospital, Vientiane, Lao PDR; Lao-Oxford-Mahosot Hospital-Wellcome Research Unit OX3 7JX (LOMWRU), Microbiology Laboratory, Mahosot Hospital, Vientiane, Lao PDR; Lao-Oxford-Mahosot Hospital-Wellcome Research Unit OX3 7JX (LOMWRU), Microbiology Laboratory, Mahosot Hospital, Vientiane, Lao PDR; Center for Tropical Medicine and Global Health, Nuffield Department of Medicine, New Richards Building, Oxford University, Oxford OX3 7LG, UK; Lao-Oxford-Mahosot Hospital-Wellcome Research Unit OX3 7JX (LOMWRU), Microbiology Laboratory, Mahosot Hospital, Vientiane, Lao PDR; Oxford Autoimmune Neurology Group, Nuffield Department of Clinical Neurosciences, Nuffield Department of Medicine, Oxford University, John Radcliffe Hospital, Oxford OX3 9DU, UK; Oxford Autoimmune Neurology Group, Nuffield Department of Clinical Neurosciences, Nuffield Department of Medicine, Oxford University, John Radcliffe Hospital, Oxford OX3 9DU, UK; Department of Neurology, Oxford University Hospitals, John Radcliffe Hospital, Oxford OX3 9DU, UK; Lao-Oxford-Mahosot Hospital-Wellcome Research Unit OX3 7JX (LOMWRU), Microbiology Laboratory, Mahosot Hospital, Vientiane, Lao PDR; Center for Tropical Medicine and Global Health, Nuffield Department of Medicine, New Richards Building, Oxford University, Oxford OX3 7LG, UK

**Keywords:** autoimmune, Laos, LGI1, meningoencephalitis, neuroimmunology, NMDAR

## Abstract

**Background:**

The importance of autoimmune encephalitis and its overlap with infectious encephalitides are not well investigated in South-East Asia.

**Methods:**

We report autoantibody testing, using antigen-specific live cell-based assays, in a series of 134 patients (cerebrospinal fluid and sera) and 55 blood donor controls (sera), undergoing lumbar puncture for suspected meningoencephalitis admitted in Vientiane, Lao People's Democratic Republic (PDR).

**Results:**

Eight of 134 (6%) patients showed detectable serum neuronal autoantibodies, against the N-methyl-D-aspartate and gamma-aminobutyric acid A receptors (NMDAR and GABA_A_R), and contactin-associated protein-like 2 (CASPR2). Three of eight patients had accompanying autoantibodies in cerebrospinal fluid (two with NMDAR and one with GABA_A_R antibodies), and in two of these the clinical syndromes were typical of autoimmune encephalitis. Three of the other five patients had proven central nervous system infections, highlighting a complex overlap between diverse infectious and autoimmune causes of encephalitis. No patients in this cohort were treated with immunotherapy, and the outcomes were poor, with improvement observed in a single patient.

**Conclusions:**

In Lao PDR, autoimmune encephalitis is underdiagnosed and has a poor prognosis. Empiric immunotherapy should be considered after treatable infectious aetiologies are considered unlikely. Awareness and diagnostic testing resources for autoimmune encephalitis should be enhanced in South-East Asia.

## Introduction

Encephalitis, inflammation of the brain, is a medical emergency with high morbidity and mortality and it predominantly occurs secondary to infectious and immune-mediated causes.^1–9^ The incidence of all-cause encephalitis has been estimated at 3–7 cases per 100 000 person-years in the UK and USA. In these regions, several aetiological descriptions of encephalitis have focused on infections, with no known cause described in approximately 60% of patients. ^1–9^ However, over the last few years, a range of neuroglial surface-directed autoantibodies has been discovered in patients with forms of autoimmune encephalitis (AE). As these autoantibodies target the extracellular domains of key neuroglial proteins, they are likely to be pathogenic in humans. The most common autoantibody targets include the N-methyl-D-aspartate receptor (NMDAR), leucine-rich glioma-inactivated 1 (LGI1), the gamma-aminobutyric acid receptor A and B receptors (GABA_A_R, GABA_B_R) and contactin-associated protein-like 2 (CASPR2).^2–5^ These autoantibodies explain a substantial proportion of previously idiopathic cases and, importantly, typically associate with immunotherapy-responsive conditions: meaning they are considered ‘not to miss’ diagnoses.^2–6^

This shift is reflected by a recent USA study, in which autoimmune aetiologies of encephalitis were described at least as frequently as infectious causes.^3^ In this study, the incidence of AE increased around threefold in a comparison of 1995–2005 with 2006–2015, likely reflecting increased awareness of AE and a growing emphasis on neuronal autoantibody testing. In contrast, the incidence of all infectious aetiologies remained stable at 1.0 per 100 000 person-years.

In addition, overlaps have been recognised between infectious and autoimmune causes of encephalitis. In particular, herpes virus simplex encephalitis (HSVE) and Japanese encephalitis (JE) have been described to precede AE associated with autoantibodies to NMDAR, GABA_A_R and others.^7–9^

Epidemiological data about meningoencephalitis in southeast Asian countries are sparse with cohorts examining its causes described in Taiwan, Thailand, China, Vietnam, Cambodia, Korea and the Lao People's Democratic Republic (Lao PDR, Laos).^10–11^ These studies show a predominance of unknown aetiologies, with infectious aetiologies only confirmed in 17–54% of patients and few reports of autoimmune encephalitis.^12–17^ Here, we present the first examples of neuronal autoantibodies and AE in patients with meningoencephalitis in Laos, describe their phenotypes and identify those with potentially novel infectious–autoimmune overlaps.

## Materials and Methods

### Patient and clinical data

A prospective study during January 2003–August 2011 recruited all consenting inpatients with suspected central nervous system (CNS) infection in Mahosot Hospital, Vientiane Capital, for whom diagnostic lumbar puncture was indicated in the opinion of the responsible physician. Patient history and examination findings were recorded on standardised forms and meningitis, meningoencephalitis and encephalitis were defined as previously described.^11,18^

Cerebrospinal fluid (CSF) and sera were aliquoted and immediately stored at −80°C. All patient samples underwent a panel of laboratory tests including full blood count, biochemistry panel, culture and serologic and molecular assays for a range of bacteria, viruses, parasites and fungi.^11^ HIV-1 and HIV-2 rapid diagnostic tests were performed when requested. CT brain scans were available starting in 2002 but were rarely used, especially for intensive care patients, because of difficulties in transferring patients. MRI and EEG were not available.

Clinical case descriptions were prepared (MM, SR, PNN) and reviewed (CEU, SRI), blind to autoantibody results, to determine whether they met 2016 consensus criteria for AE (Table 1).^19^

**Table 1.** Clinical and paraclinical features of patients with encephalitis-associated autoantibodies in Laos

		IgG-specificity and endpoint titres							
Patient age (y) and gender	Clinical features and CNS syndrome	Serum	CSF	CSF WBC (cells/uL)	CSF: serum glucose ratio	CSF protein (mg/dL)	Identified infection	Outcome	Meeting criteria for possible autoimmune encephalitis?^19^
A. 22M	Prodromal fever then acute confusion, neck stiffness, agitation, delirium, hypersomnolence/insomnia, catatonia; meningoencephalitis	NMDAR 1:200	NMDAR 1:200	15	0.50	55	None	Discharged, not improved - diagnosis catatonic schizophrenia	YES
B. 45F	Prodromal fever, headache, neck stiffness then acute confusion, drowsiness, agitation, delirium; admitted initially to psychiatry; meningoencephalitis	NMDAR 1:500	NMDAR 1:2	50	0.81	28	None	Discharged moribund	YES
C. 35M	7-d history of fever and headache followed by acute confusion, drowsiness; meningoencephalitis	NMDAR 1:500	Neg.	85	0.35	55	Pus in ears, culture negative	Deceased	NO
D. 53M	10-d history of fever, headache, neck stiffness, cough, vomiting, confusion, right leg weakness; meningoencephalitis	NMDAR 1:400	Neg.	610	CSF 2.0 mmol/L*	276	TB culture CSF positive	Deceased, diagnosis TB meningitis	NO
E. 45M	Acute onset of fever, headache, neck stiffness, agitation, drowsiness, confusion, with single convulsion; meningoencephalitis	NMDAR 1:200	Neg.	10	0.43	38	Serum murine typhus IgM+, *Rickettsia typhi* PCR negative	Discharged well	NO
F. 73M	Acute onset of fever, neck stiffness, delirium, agitation and altered consciousness; admitted initially to psychiatry; meningoencephalitis	NMDAR 1:200	Neg.	5	CSF 5.2 mmol/L*	30	None	Discharged moribund	NO
G. 51M	1-mo weight loss preceding acute onset fever, headache and neck stiffness; meningitis; suspected underlying malignancy	CASPR2 1:800	Neg.	0	1.09	72	*N. meningitidis* PCR positive on CSF (culture negative)	Discharged AMA, no improvement	NO
H. 57M	7-d fever, headache, cough and rash without seizures or confusion	GABA_A_R 1:160	GABA_A_R 1:8	18	0.34	20	None	Unknown	NO

Abbreviations: AMA, against medical advice; CASPR2, contactin-associated protein-like 2; CSF, cerebrospinal fluid; GABA_A_R, gamma-amino butyric acid A receptor; NMDAR, N-methyl-D-aspartate receptor; WBC, white blood cells.

The following cut-offs were used for positive serum neuronal autoantibody titres: NMDAR antibodies >1:100, CASPR2 antibodies >1:500, GABA_A_R antibodies >1:50. In CSF, any antigen-specific reactivity is considered positive.

*no concurrent serum glucose available for these patients.

See Dubot-Pérès et al. (2019)^11^ for diagnostic techniques for the pathogens given in the Identified infection column.

The criteria for possible autoimmune encephalitis used are = diagnosis can be made when all three of the following criteria have been met: (a) subacute onset (rapid progression of less than 3 mo) of working memory deficits (short-term memory loss), altered mental status or psychiatric symptoms; (b) at least one of the following: new focal CNS findings, seizures not explained by a previously known seizure disorder, CSF pleocytosis (white blood cell count of more than five cells per mm^3^), MRI features suggestive of encephalitis; and (c) reasonable exclusion of alternative causes.^19^

### Autoantibody testing

Data on the infectious disease aetiologies have been published.^11^ Autoantibodies were tested in available CSF and serum samples from 134 patients: 92 patients without an identified infectious aetiology and 42 patients with a confirmed infectious diagnosis. In addition, sera from 55 healthy blood donor controls from Vientiane were tested. Samples were tested on antigen-specific live cell-based assays, against LGI1, CASPR2 and the NMDA and GABA_A_ receptors, the commonest neuroglial surface-directed autoantibodies encountered in clinical practice, as previously described.^20–22^ Positive results were titrated to endpoint dilutions.

## Results

### Clinical findings

From the 134 paired CSF/serum samples, 42 (31%) had a confirmed infectious aetiology that included Japanese encephalitis virus (JEV; n=7), *Cryptococcus* spp. (n=7), *Mycobacterium tuberculosis* (n=5), Herpes simplex virus (n=5), Dengue virus (n=4), *Streptococcus pneumoniae* (n=3), *Rickettsia typhi* (n=3), *Orientia tsutsugamushi* (n=3), *Neisseria meningitidis* (n=2), *Streptococcus suis* (n=2) and *Leptospira* spp. (n=1), and two had suspected infectious aetiologies (JEV n=1, mumps/measles n=1).^11^ The remaining 90 patients had meningoencephalitis of unknown aetiology. The 55 healthy controls had a median (range) age of 19 (17–19) y and 16.4% were female.

### Patients with autoantibodies

**Figure 1.**
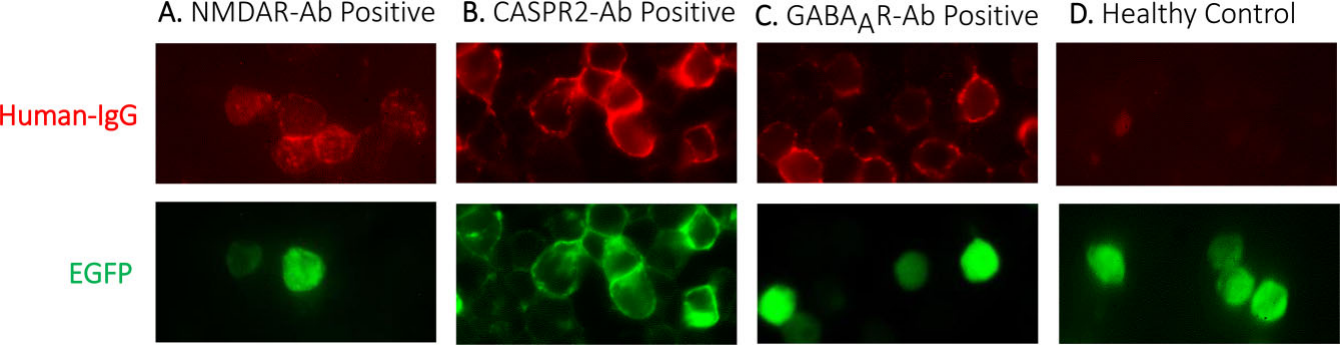
Live cell-based assays in antigen-expressing HEK293T cells demonstrate surface binding of autoantibodies from the serum of patients with suspicion of CNS infection in Laos. Autoantibodies shown against the (A) N-methyl-D-aspartate receptor (NMDAR), (B) contactin-associated protein-like 2 (CASPR2) and (C) gamma-amino butyric acid A receptor (GABA_A_R). All at 100x magnification. (D) Absence of staining shown in the serum of a representative healthy control, using NMDAR-transfected HEK293T cells. For each assay, CNS autoantibody staining is shown in red and co-transfected or antigen-tagged enhanced green fluorescent protein (EGFP) in green.

Overall, none of the 55 healthy controls but 8/134 (6%) patients had detectable neuroglial surface autoantibodies (Table 1 and Figure 1); 0/8 fulfilled definite AE criteria, lacking MRI and EEG findings, which are part of the formal diagnostic requirements. These eight patients were aged 0.08–77 (median 32) y and one was female. These demographics were similar to the overall seronegative case series, in which 44/126 (35%) were female and the median age was 45 (22–73) y. Six of eight seropositive patients had serum NMDAR autoantibodies (endpoint dilutions from 1:200 to 1:500). In two (patients A and B), the CSF also harboured NMDAR antibodies with serum:CSF NMDAR antibody ratios of 1 and 250. Both these patients had syndromes compatible with definite NMDAR antibody encephalitis (and fulfilled the criteria for possible AE) and were discharged with catatonic schizophrenia and moribund, respectively.

Two other seropositive patients had either serum CASPR2 antibodies (without CSF CASPR2 antibodies, patient G) or serum GABA_A_R antibodies with accompanying CSF GABA_A_R antibodies (patient H). The latter patient had a serum:CSF autoantibody ratio of 20, indicative of marked intrathecal synthesis of the GABA_A_R antibodies, with a meningoencephalitis syndrome but no seizures.

Six of the eight patients had neck stiffness, consisting of 5/6 with NMDAR antibodies and one with CASPR2 antibodies (Table 1). Six patients with suspected CNS infection were classified as having meningoencephalitis, one as having meningitis (patient G) and one (patient H) did not fulfil WHO encephalitis or meningitis case definitions.^18^ Of the 134 patients tested for autoantibodies, 76 (57%) met WHO criteria for encephalitis and 34 (26%) met WHO criteria for meningitis.

### Infectious overlaps

As described in Table 1, two of the eight seropositive patients had definite infectious diagnoses (*M. tuberculosis* in patient D and *N. meningitidis* in patient G) with serum, but not CSF, NMDAR and CASPR2 antibodies, respectively. These findings of an alternative aetiology exclude them from criteria-based diagnoses of AE. Another patient had serum NMDAR antibodies and anti-*R. typhi* IgM (patient E) detected but a negative peripheral blood EDTA buffy coat PCR for *R. typhi*, leading to uncertainty with the diagnosis of acute murine typhus.^23^ One patient (patient C) had a moderate (1:500) serum NMDAR antibody level and purulent discharge from the ears with a history of chronic otitis media; hence, a primary infectious aetiology from contiguous spread was considered more likely than a primary autoimmune condition.

### Outcomes

None of the eight patients with autoantibodies received immunotherapy, including adjunctive corticosteroids for infectious disease indications. At last follow-up at a median (range) interval of 10 (2–40) d, two were deceased, two discharged moribund, two discharged without improvement and one was in an unknown clinical condition. Only one patient (13%) improved during their hospitalisation (Table 1).

## Discussion

We provide the first description of neuronal autoantibodies in patients in Laos. Although limited clinical and investigation data were available in these patients, our findings suggest that cases of AE in this population likely remain undiagnosed and untreated. This observation may apply to many other countries where widespread autoantibody testing is not available, and infectious diagnoses are historically considered more frequent. Yet, autoimmune causes of encephalitis appeared as common as infectious aetiologies in adjacent Thailand.^12^

Of interest, we detected moderate-titre autoantibodies, absent from the healthy controls tested, which target the extracellular domains of key neuronal proteins, exclusively in the serum of the small subset of patients with proven infectious aetiologies, namely, *M. tuberculosis* and *N. meningitidis*. These findings extend the possible infections that may be associated with neuronal autoantibodies, beyond the recognised prodromal associations with HSVE and JE.^7–9^ Future studies should systematically and longitudinally evaluate the evolution of these reactivities in diverse infectious settings. We hypothesise that such CNS infections may expose CNS antigens, which drain through meningeal lymphatics to stimulate peripheral autoantibody-reactive B cells,^24^ with cervical lymph nodes representing the most likely site for initiation of this reaction. CSF autoantibodies are highly specific in the diagnosis of these conditions and their absence in the CSF of these patients supports that they are likely an epiphenomenon of infection rather than due to a primary autoimmune process.

The limitations of the study include the relatively small sample size, that patients were from a single centre, the lack of MRI and EEG data and investigations for systemic tumours associated with autoantibodies and the absence of testing by a ‘confirmatory’ detection method of rodent brain section immunohistochemistry due to insufficient sample volumes.^25^ Nevertheless, the exclusive binding of any serum/CSF sample to a single autoantigen confirms a significant degree of specificity.^26^

Neck stiffness is unusual in AE but 6/8 patients described here had neck stiffness, including one patient meeting WHO criteria for meningitis but not encephalitis.^11,18^ This suggests that future studies should search for autoantibodies in patients with meningitic presentations.^27^ Further, CNS autoantibodies were detected in one patient who did not fulfil either WHO encephalitis or meningitis case definitions.

These data, plus several emerging reports, collectively suggest that AE is not uncommon worldwide.^3,19^ AE results in significant morbidity and mortality but usually responds to immunotherapies. Hence, the trajectory and outcome of these patients can be significantly improved with appropriate recognition and care. Indeed, clinical recognition of AE is key: individual forms show distinctive clinical features such as their psychopathological features,^28^ profile of cognitive deficits,^29^ phenomenology of movement disorders^30^ and highly characteristic seizure semiologies.^26,31^ Accurate identification of these features, in parallel with autoantibody testing, permits rational administration of immunotherapies. Engagement with general, infectious disease, neurology and psychiatry health workers and policymakers will be essential. Some AE patients are given a variety of alternative diagnoses that include delirium, functional disorders and catatonia. Indeed, one patient in this series with catatonia and CSF NMDAR antibodies was likely to have been misdiagnosed with this primary psychiatric condition.^28^

In mainland South-East Asia, as far as we are aware, laboratory diagnosis of AE is only available in Thailand, Vietnam, Singapore and Malaysia, at very few specialised centres. Autoantibody detection utilises simple 2–3 colour fluorescent microscopy, already widely available within South-East Asia for diagnosis of infectious diseases such as rabies, with trained diagnostic staff and systems that could be repurposed for AE diagnosis. Creation of laboratory networks for the diagnosis of AE, using existing equipment and staff trained in indirect immunofluorescence assays, would raise awareness, increase our understanding of AE risk factors, epidemiology and the infectious disease–autoimmune interface and provide key information for the optimal management of individual patients.

Immunotherapies such as intravenous immunoglobulin, plasmapheresis, azathioprine, mycophenolate, cyclophosphamide and monoclonal antibody therapy (e.g. rituximab, tocilizumab) may be prohibitively expensive or unavailable in resource-poor settings.^4^ However, corticosteroids are widely available and highly effective in many patients with AE.^5,6^ In Vientiane, only corticosteroids and cyclophosphamide were available medications as of January 2021. Adverse consequences of lone corticosteroid therapy in some conditions that are relatively common in South-East Asia (e.g. *M. tuberculosis* CNS infection) mean careful consideration will be needed to exclude these infections. In addition, surgical resection of an underlying tumour can improve outcomes in patients with paraneoplastic AE, and such operations can be performed in many South-East Asian hospitals.

Taken together, our study suggests that a proportion of patients in Laos with meningoencephalitis and poor outcomes have a treatable form of AE. Others represent potentially novel infectious–autoimmune overlaps, an area for future study. Recognition of AE may remain relatively neglected in much of the world and should be promoted with appropriate clinical training and autoantibody testing facilities. The risk factors and epidemiology in these regions may provide important insights for the optimal management of individual patients worldwide.

## Data Availability

The data underlying this article will be shared on reasonable request to the corresponding author.
